# *CDCA7* Facilitates Tumor Progression by Directly Regulating *CCNA2* Expression in Esophageal Squamous Cell Carcinoma

**DOI:** 10.3389/fonc.2021.734655

**Published:** 2021-10-19

**Authors:** Hongyi Li, Yongjia Weng, Shaojie Wang, Fang Wang, Yanqiang Wang, Pengzhou Kong, Ling Zhang, Caixia Cheng, Heyang Cui, Enwei Xu, Shuqing Wei, Dinghe Guo, Fei Chen, Yanghui Bi, Yongsheng Meng, Xiaolong Cheng, Yongping Cui

**Affiliations:** ^1^ Department of Pathology & Shanxi Key Laboratory of Carcinogenesis and Translational Research of Esophageal Cancer, Shanxi Medical University, Taiyuan, China; ^2^ Key Laboratory of Cellular Physiology, Ministry of Education, Shanxi Medical University, Taiyuan, China; ^3^ Department of Pathology, the First Hospital, Shanxi Medical University, Taiyuan, China; ^4^ Department of Pathology, Shanxi Province Cancer Hospital, Taiyuan, China; ^5^ Department of Thoracic Surgery (Ⅰ), Shanxi Province Cancer Hospital, Taiyuan, China; ^6^ The Science Research Center, Shanxi Bethone Hospital, Taiyuan, China; ^7^ Tumor Biobank, Shanxi Province Cancer Hospital, Taiyuan, China

**Keywords:** *CDCA7*, cell cycle, *CCNA2*, copy number amplification, ESCC (esophageal squamous cell carcinoma)

## Abstract

**Background:**

*CDCA7* is a copy number amplified gene identified not only in esophageal squamous cell carcinoma (ESCC) but also in various cancer types. Its clinical relevance and underlying mechanisms in ESCC have remained unknown.

**Methods:**

Tissue microarray data was used to analyze its expression in 179 ESCC samples. The effects of *CDCA7* on proliferation, colony formation, and cell cycle were tested in ESCC cells. Real-time PCR and Western blot were used to detect the expression of its target genes. Correlation of *CDCA7* with its target genes in ESCC and various SCC types was analyzed using GSE53625 and TCGA data. The mechanism of *CDCA7* was studied by chromatin immunoprecipitation (ChIP), luciferase reporter assays, and rescue assay.

**Results:**

The overexpression of *CDCA7* promoted proliferation, colony formation, and cell cycle in ESCC cells. *CDCA7* affected the expression of cyclins in different cell phases. GSE53625 and TCGA data showed *CCNA2* expression was positively correlated with *CDCA7*. The knockdown of *CCNA2* reversed the malignant phenotype induced by *CDCA7* overexpression. Furthermore, *CDCA7* was found to directly bind to *CCNA2*, thus promoting its expression.

**Conclusions:**

Our results reveal a novel mechanism of *CDCA7* that it may act as an oncogene by directly upregulating *CCNA2* to facilitate tumor progression in ESCC.

## Background

Esophageal cancer which accounts for 11% of diagnosed cancers was the fourth most common cancer type. In China, the dominant histologic type of esophageal cancer is esophageal squamous cell carcinoma (ESCC) which causes more than 175,000 deaths every year (1). The 5-year survival rate of ESCC which ranges from 22% to 30% still tends to be low because of the limitation of technical developments for early diagnosis and treatment (2). However, the advent and progression of next-generation sequencing (NGS) in recent years has given us some achievements on ESCC (3–7).

In our previous WGS analysis of 31 ESCC tumor tissues and matched adjacent non-tumor tissues, we identified some genes with copy number variation, including cell division cycle-associated 7 gene (*CDCA7*), that was amplified in 5 out of 31 ESCC patients (8). *CDCA7* is located on 2q31.1. It is characterized as a c-Myc and E2F responsive gene that participates in neoplastic transformation (9, 10). It has been reported that the expression of *CDCA7* is elevated in a high fraction of human lung, colon, ovary, rectum, stomach, and uterus cancer types, suggesting that *CDCA7* may play a crucial role in cancer development (11–13). A recent study showed that the high expression of *CDCA7* predicted poorer disease-free survival in patients with triple-negative breast cancer (TNBC) and was associated with metastatic relapse status (14). One research in lung adenocarcinoma reported that *CDCA7* promoted lung adenocarcinoma proliferation through regulating the cell cycle, while its mechanism has not been completely elucidated yet (15). Meanwhile, *CDCA7* as a DNA-binding protein can function as a transcription regulator to mediate the tumor-promoting effect (9).

CCNA2, which is synthesized at the beginning of S-phase (16, 17), binds and activates cyclin-dependent kinases (CDK) CDK2 and CDK1, the catalytic partners of CCNA2. The CDK2/CCNA2 complex is the machinery that drives the progression of S-phase. In the S-phase of the cell cycle, the CCNA2–CDK complex can phosphorylate key substances in the process of DNA replication, such as CDC6. This phosphorylation is crucially important for the initiation of DNA replication. It is possible that CCNA2–CDK contributes to tumorigenesis by the phosphorylation of oncoproteins and the increased expression level of *CCNA2* accelerates cell proliferation once the tumor has formed (18–20). Increased expression of *CCNA2* has been observed in various types of cancer such as lung, breast, liver, cervical, and others (18, 21–24). The expression level of *CCNA2* is closely related with cell proliferation; thus, it is used as a proliferation marker for the molecular diagnosis of cancer (18). Meanwhile, the expression of *CCNA2* appears to be of prognostic value for the prediction of survival and early relapse in many types of cancer (18, 25).

In our study, we analyzed the copy number amplification data from The Cancer Genome Atlas (TCGA) database in various types of tumors and the correlation between *CDCA7* expression level and clinical variables in ESCC using the mRNA expression data from the GEO database. Furthermore, we verified that *CDCA7* has as a tumor-promoting role in ESCC, and elaborated on its potential mechanisms of carcinogenesis. Our results show that CDCA7 may bind to *CCNA2* to upregulate its expression. Therefore, increased CCNA2 promotes the proliferation of ESCC cells, thus promoting tumor growth. Our study provides useful clues for more effective therapeutic strategies against ESCC.

## Methods

### Clinical Samples

The copy number data were obtained from our study. The tumor and the matched adjacent non-tumor samples were recruited from Shanxi Cancer Hospital of Shanxi Medical University. The patients were without preoperative chemotherapy, radiotherapy, and other treatments before operation, and written consent was obtained from all of them. Hematoxylin and eosin (H&E) staining was used to diagnose these tissues, and the diagnosis was performed by at least two pathologists independently. The ESCC individuals were staged according to the American Joint Commission for Cancer (AJCC)/International Union Against Cancer (UICC) TNM staging system (eighth edition). The study was approved by the Institutional Reviewing Board (IRB) and the Research Committee of Shanxi Medical University.

### Cell Lines and Cell Culture

ESCC cell lines KYSE150, KYSE180, KYSE450, and TE-1 and immortal embryonic esophageal epithelium cell lines NE3 and HET-1A used in the research were purchased from the Cell Bank of Type Culture Collection of the Chinese Academy of Sciences. The cell line 293T was from our lab. The cell lines KYSE150, KYSE180, KYSE450, and TE-1 were cultured in HyClone™ RPMI-1640 medium, and the cell lines HET-1A and 293T were cultured in HyClone™ DMEM/High Glucose medium (GE Healthcare Life Sciences, HyClone Laboratories, Logan, UT, USA). The culture was with 10% fetal bovine serum (FBS; Gibco; Thermo Fisher Scientific, Inc., Waltham, MA, USA). The cell line NE3 was cultured in a 1:1 mixture of EpiLife medium (Cascade Biologics, Inc., Portland, OR, USA) and defined keratinocyte serum-free medium (dKSFM; Gibco; Thermo Fisher Scientific, Inc., Waltham, MA, USA). All of the cell lines were cultured at 37°C, 5% CO_2_. The culture medium was replaced according to the cell state. Subculture was carried out when the cell fusion was about 80%–90%.

### Overexpression and Knockdown of *CDCA7* in ESCC Lines

SiRNAs or plasmids were transfected into the cells at the logarithmic growth phase using Lipofectamine 2000 reagent (Invitrogen, Carlsbad, CA) according to the instructions of the manufacturer. For knockdown of endogenous *CDCA7*, we used vectors containing the sequence 5′-GCCCTCAGAGAATTCTGTGACTGAT-3′ (*CDCA7*-si1) and 5′-CATCCGTGACCCTTCCGCATATAAT-3′ (*CDCA7-*si2). These shRNAs were cloned into the vector pHBLV-U6-Scramble-ZsGreen-Puro vector. For stable overexpression, the coding sequence (CDS) region of the *CDCA7* gene was cloned into pHBLV-CMV-MCS-3FLAG-EF1-ZsGreen-T2A-PURO. The recombinant plasmids and the packaging plasmids (Hanbio Biotechnology Co., Ltd., Shanghai, China) were co-transfected into 293T cells. The lentivirus supernatant was used to infect the KYSE150, KYSE450, and KYSE180 cell lines. The negative control was the corresponding empty vectors. The KYSE150 and KYSE450 knockdown stable cell lines and the KYSE180 overexpression stable cell line were screened out for 7–14 days with 1.0, 1.0, and 0.8 µg/ml puromycin (Invitrogen; Thermo Fisher Scientific, Inc.), respectively. The efficiency of knockdown and overexpression was determined by real-time PCR and Western blot assay. We used small interference RNA (siRNA) for *CCNA2* knockdown, and the siRNA sequence information is as follows: *CCNA2*-si1, 5′-CTATGGACATGTCAATTGT-3′; *CCNA2*-si2, 5′-GAGTGTTAATGAAGTACCA-3′. The CDS of *CDCA7* and *CCNA2* genes was cloned into the pcDNA3.1 vector with a V5 tag and 6*His tag.

### MTT Assay

The MTT assay was performed using a 96-well plate with 5 × 10^3^ transfected cells each well and cultured for 24–120 h. A 20-µl MTT solution (5 mg/ml) was added to a 200-µl culture medium each well for 4 h at 37°C. The MTT formazan crystals that remained after removing the medium were then solubilized in dimethyl sulfoxide (DMSO) for 15–20 min. The absorbance was measured by a spectrophotometer at 490 nm to show the relative number of surviving cells in each well indirectly.

### Colony Formation Assay

A total of 1,000 cells/well were seeded into six-well plates and incubated at 37°C and 5% CO_2_ for 10–15 days. Polyformaldehyde (4%) was used to fix these cells and 1% crystal violet was used to stain these cells subsequently. The numbers of colonies containing more than 10 cells were counted.

### Flow Cytometry Analysis

Cells collected were fixed with 70% alcohol and stored overnight at −20°C. Propidium iodide (PI) was used to stain the collected cells according to the instructions of the manufacturer. The stained cells were analyzed using a flow cytometer (BD Company, USA).

### Immunofluorescence

KYSE150, KYSE180, and KYSE450 cells were transfected with CDCA7-V5 plasmid and empty vector, respectively. Formaldehyde (4%) was used to fix the cells for 10 min. BSA (1%) was used to incubated the cells for 1 h to block non-specific protein–protein interactions after permeabilized by 0.1% Triton X-100. The cells were incubated with the primary antibody rabbit anti-V5 (Abcam, Cambridge, UK, 2 µg/ml) overnight at 4°C. Alexa Fluor^®^ 594 goat anti-rabbit IgG antibody (Thermo Fisher, Carlsbad, USA, 1:1,000) was used for 30 min at room temperature after washing four times in PBS. DAPI at a concentration of 0.5 µg/ml was used to stain the cell nuclei.

### Chromatin Immunoprecipitation Sequencing Assay

KYSE150 cells were transfected with the V5-tagged CDCA7 plasmid for the chromatin immunoprecipitation (ChIP) assay. The assay was performed according to the instructions of the manufacturer (Millipore, Burlington, MA, USA). The DNA fragments were enriched by anti-V5 antibody (Abcam, Cambridge, UK), and the isotype IgG (Abcam, Cambridge, UK) was used as a negative control. CHIP-seq was performed by Novogene (Beijing, China). Screening and quality control of the CHIP-seq were based on standard protocol. The sequences of primers used for the amplification of *CCNA2* genome regions containing a putative CDCA7 binding site are listed in **Table S1**.

### Western Blot

The cells were lysed for 1 h with RIPA buffer containing protease and phosphatase inhibitors (Thermo Fisher Scientific) on ice. The components of RIPA buffer are as follows: 1% Triton X-100, 50 mM Tris–HCl, pH 7.6, 150 mM NaCl, 1% sodium deoxycholate, and 0.1% SDS. The lysates were centrifuged at 12,000*g* at 4°C for 30 min, and the total protein concentrations of supernatant were determined by the Bradford method. Fifty micrograms of protein was separated by 10% SDS-PAGE and then transferred onto polyvinylidene fluoride (PVDF) membranes (Millipore, USA). The membrane was incubated with special antibodies, including CDCA7, CCND1, CCNA2, CCNE1, and GAPDH, at 4°C overnight. The IRDye 800CW secondary antibody (Abcam, Cambridge, UK) was used to detect the blot. A relative amount of protein was normalized to GAPDH level. The antibodies used in this experiment are shown as follows: CDCA7 (Sigma, USA), CCND1 (Proteintech, Rosemont, IL, USA), CCNA2 (Proteintech, Rosemont, IL, USA), CCNE1 (Proteintech, Rosemont, IL, USA), and GAPDH (Proteintech, Rosemont, IL, USA).

### RNA Extraction and Real-Time PCR

Total RNA of ESCC cells was purified using RNAiso plus (Takara, Dalian, China). Two micrograms of total RNA was used for complementary DNA (cDNA) synthesis using a PrimeScript^®^ RT reagent kit with gDNA Eraser (Takara). TB Green^®^ Premix Ex Taq^®^ II kit (Takara) was used in real-time PCR according to the instruction of the manufacturer. All real-time PCR reactions were performed in triplicate with an Applied Biosystems Step One Plus (ABI, Foster City, CA, USA). The relative expression levels of the target genes were normalized to endogenous GAPDH. Quantification of the expression levels of target genes was calculated using the 2^−ΔΔCt^ formula. The primers synthesized by Thermo Fisher are listed in **Table S2**.

### Dual-Luciferase Reporter Assay

According to the results of ChIP-seq, we cloned the CDCA7 DNA-binding fragment with *CCNA2* from the KYSE150 cell line genomic DNA. The cloned DNA fragment was constructed into the reporter plasmid of pGL3-promoter (Promega, Madison, WI, USA). Then, we divided this DNA fragment into four segments and constructed them into the pGL3-promoter vector. The different DNA fragments were cloned using the primers listed in **Table S3**. Six motif sequences obtained from ChIP-seq were also constructed into pGL3-promoter vector. Cells (3 × 10^4^) were cultured in triplicate in 24-well plates for 12–24 h. Then, the pGL3 reconstruction reporter plasmids were transiently co-transfected with the pRL-TK plasmid into KYSE150 and KYSE150 knockdown cells using Lipofectamine 2000 reagent (Invitrogen, Carlsbad, CA). After transfection for 48 h, luciferase and Renilla signals were measured according to the instruction of Dual-Luciferase Reporter Assay Kit (Promega, Madison, USA).

### Mouse Xenograft Assay

The effects of *CDCA7* on tumorigenesis and growth *in vivo* were detected *via* mouse xenograft assay. We used 20 5- to 6-week-old female NU-Foxn1^nu^ nude mice (Vital River Laboratory Animal Technology Co., Ltd., Beijing, China) for the mouse xenograft assay. A total of 3 × 10^6^ KYSE150-NC cells or *CDCA7*-knockdown stable KYSE150 cells were used to inject into the right or left oxter of female NU-Foxn1^nu^ nude mice, respectively. Tumor size and weight were determined with calipers and balance twice a week. The mice were executed and the tumors were removed after 28 days. The formula *V* = (*W* ^2^ × *L*)/2 was used to calculate the tumor volume. *V* is the tumor volume, *W* is the tumor width, and *L* is the tumor length. Tumor size was presented as mean ± standard deviation (SD).

### Immunohistochemistry

The isolated xenograft tumor tissues were fixed using formalin and embedded by paraffin for immunohistochemical staining. Briefly, xylene and a series of grades of alcohol were used to deparaffinize and rehydrate these sections, and the sections were then soaked with 3% H_2_O_2_ 15 min. Sodium citrate buffer (pH 6.0) or Tris-EDTA buffer (pH 9.0) were used for antigen retrieval for 4 or 3 min in a pressure cooker, followed by incubation with primary antibody at 4°C overnight. The slides were incubated with second antibody at 37°C for 20 min after washing with PBS and then stained with DAB and counterstained with hematoxylin. The expression of CDCA7, Ki-67, and CCNA2 was quantitatively analyzed with Aperio Cytoplasma 2.0 software by immunohistochemistry. The antibodies used in this experiment are shown as follows: CDCA7 (Sigma, USA, 1:200 dilution), Ki-67 (Abcam, Cambridge, UK), and CCNA2 (Proteintech, Rosemont, IL, USA, 1:2,000 dilution).

### Bioinformatics and Data Analysis

The mRNA expression data and the clinical information of 179 ESCC tissues and paired non-tumor tissues by microarray analysis were from a previous study by Li et al. (26) and downloaded from the GEO database (GSE53625). The copy number data were obtained from our other study.

The copy number data of *CDCA7* in varied cancer types, including ESCC, ECA, lung squamous cell carcinoma (LUSC), head and neck squamous cell carcinoma (HNSC) collected from the TCGA database, were downloaded *via* cBioPortal for Cancer Genomics (https://www.cbioportal.org/) (27, 28). The expression data of *CDCA7* and *CCNA2* in different cancer types such as ESCC, LUSC, HNSC were downloaded from TCGA *via* Xena Browser (https://xenabrowser.net/heatmap/).

### Statistical Analyses

Each of the experiment in the study was performed in triplicate, and data were presented as the mean ± SEM. Statistical Package for Social Science for Windows (SPSS, version 20.0; IBM Inc., USA) was used to analyze the experimental data. The means of two groups and more than two groups were compared using Student’s *t*-test and one-way ANOVA, respectively. *P*-value of <0.05 was considered to be statistically significant. GraphPad Prism software was used to analyze the correlations between *CDCA7* and *CCNA2* using non-parametric correlation (Spearman).

## Results

### *CDCA7* Was Frequently Amplified in ESCC

**Figure 1:**
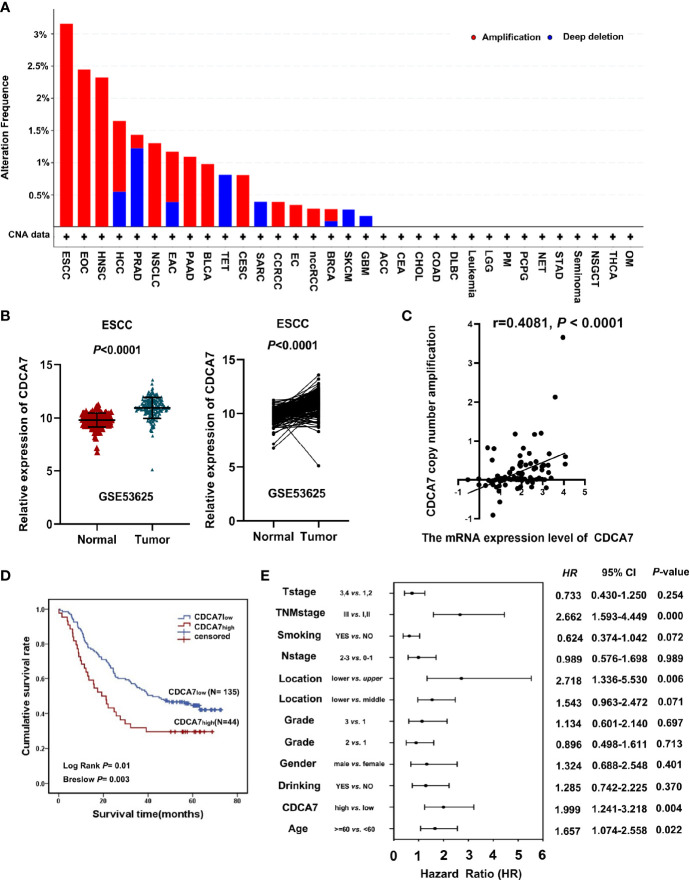
*CDCA7* expression predicts the prognosis of esophageal squamous cell carcinoma (ESCC) patients. **(A)** The *CDCA7* copy number amplification of various kinds of tumor in the TCGA database. **(B)** ESCC tumor tissues had a high *CDCA7* expression compared with its non-tumor tissues using non-paired *t*-test and paired *t*-test; *P* < 0.001. **(C)** The correlation analysis between *CDCA7* copy number amplification and expression (*r* = 0.4081, *P* < 0.0001). **(D)** The patients with *CDCA7*_low_ had better survival than those with *CDCA7*_high_ (log rank *P* = 0.01) using Kaplan–Meier survival analysis. **(E)** Multivariate analysis showed that TNM stage [hazard ratio (HR) = 2.662, 95% CI: 1.593–4.449, *P* < 0.001), location (lower vs. upper) (HR = 2.718, 95% CI: 1.336–5.530, *P* = 0.006), age (HR = 1.657, 95% CI: 1.074–2.558, *P* = 0.022), and *CDCA7* expression were independent predictive factors for overall survival (HR = 1.999, 95% CI: 1.241–3.218, *P* = 0.004).

In our previous study, *CDCA7* was identified as one of the copy number amplification genes in ESCC (8). Here, we analyzed the copy number amplification data from TCGA through cBioPortal and found that the copy number amplification of *CDCA7* existed in various kinds of tumor. Its alteration frequency was much higher in ESCC than in other tumors (**Figure 1A**). Furthermore, we analyzed the mRNA expression data of 179 pairs of ESCC tumors and adjacent normal tissues *via* microarray analysis. The mRNA expression data and the clinical information of 179 ESCC tissues and paired non-tumor tissues by microarray analysis were from a previous study by Li et al. and downloaded the from the GEO database (GSE53625) (26). We observed that *CDCA7* showed statistically higher expression levels in most of the individuals compared with that of normal tissues (**Figure 1B**). After analyzing the copy number amplification and expression of *CDCA7* in 95 ESCC patients in the TCGA database, we found that there was a correlation between the expression of *CDCA7* and the copy number amplification, indicating that the *CDCA7* copy number amplification may cause to increase its expression (**Figure 1C**).

The cohort of 179 patients was divided into two groups according to the expression level of *CDCA7*. The top 25% of patients were defined as the patients with a higher level (named as *CDCA7*_high_) and the remaining 75% were defined as the patients with a lower level (named as *CDCA7*_low_) according to the expression level of *CDCA7* from high to low. Then, we analyzed the correlation between the expression of *CDCA7* and the clinical variables in ESCC. The results in **Table 1** show that the expression of *CDCA7* was related to the grade of ESCC patients (*P* = 0.0083). The patients with *CDCA7*_high_ had a poor grade compared with the *CDCA7*_low_ patients. Furthermore, the patients with *CDCA7*_high_ had a worse survival than those with *CDCA7*_low_ (log rank *P* = 0.01, **Figure 1D**) using Kaplan–Meier survival analysis. The multivariate analysis showed that TNM stage [hazard ratio (HR) = 2.662, 95% CI: 1.593–4.449, *P* < 0.001], location (lower vs. upper) (HR = 2.718, 95% CI: 1.336–5.530, *P* = 0.006), age (HR = 1.657, 95% CI: 1.074–2.558, *P* = 0.022), and *CDCA7* expression (HR = 1.999, 95% CI: 1.241–3.218, *P* = 0.004) were independent predictive factors for overall survival (**Figure 1E**). Furthermore, *CDCA7* was related with the survival status in patients in the male group (*P* = 0.001), age <60 group (*P* = 0.02), drinking group (*P* < 0.001), smoking group (*P* = 0.014), T1+T2 group (*P* = 0.019), N0+N1 group (*P* = 0.026), and TNM stage = III group (*P* = 0.025) (**Figures S1, S2**). Hence, we speculate the copy number amplification and high expression level of *CDCA7* may promote the occurrence and development of ESCC.

**Table 1:** Correlation analysis between *CDCA7* copy number in ESCC and clinicopathological variables.

Clinical features	Total (*n* = 179)	*CDCA7*_High_ (*n* = 44)	*CDCA7*_Low_ (*n* = 135)	*P*-value
**Age**				
<60	91	24	67	0.571
≥60	88	20	68	
**Gender**				
Female	33	10	23	0.398
Male	146	34	112	
**Location**				
Upper	20	5	15	0.794
Middle	97	22	75	
Lower	62	17	45	
**Smoking**				
Never	65	15	50	0.724
Yes	114	29	85	
**Drinking**				
Never	73	17	56	0.739
Yes	106	27	79	
**Grade**				
Well	32	6	26	**0.008**
Moderately	98	18	80	
Poorly	49	20	29	
**T stage**				
1 + 2	39	12	27	0.310
3 + 4	140	32	108	
**LN stage**				
N0–N1	145	34	111	0.467
N2–N3	34	10	24	
**TNM stage**				
1 + 2	87	23	64	0.576
3 + 4	92	21	71	

In bold: P<0.05 was considered to be statistically significant.

### *CDCA7* Promotes Cell Proliferation, Colony Formation, and Cell Cycle of ESCC Cells

**Figure 2:**
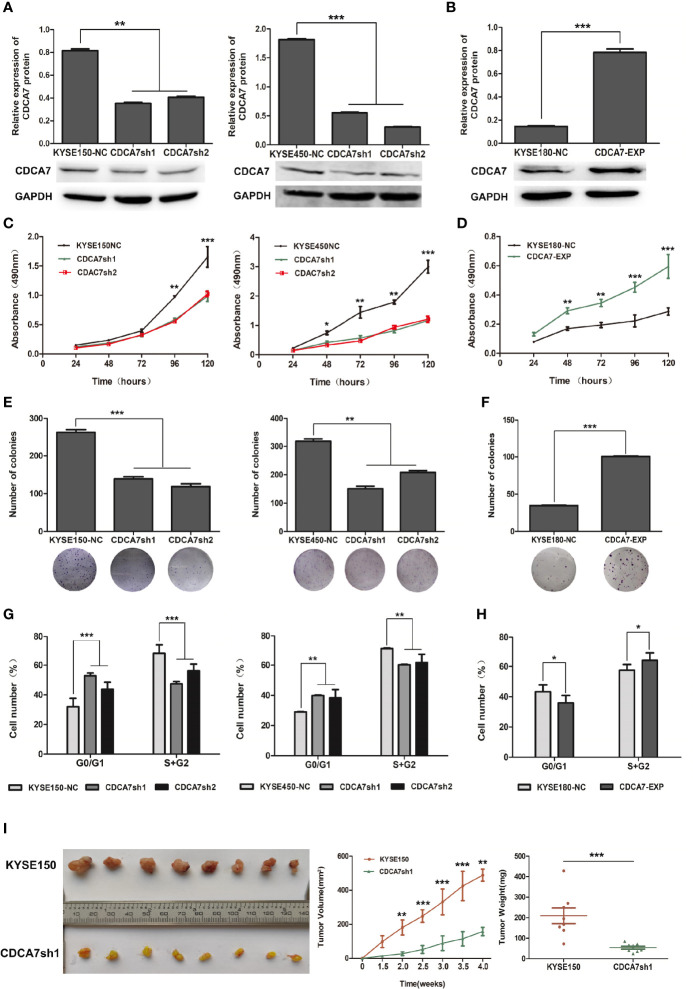
The effect of *CDCA7* gene in ESCC cell lines. **(A)** The *CDCA7* knockdown efficiency in KYSE150 and KYSE450 cells. **(B)** The *CDCA7* overexpression efficiency in KYSE180 cells. **(C)** *CDCA7* knockdown inhibited the proliferation of ESCC Cells. **(D)** *CDCA7* overexpression promoted the proliferation of ESCC cells. **(E)** *CDCA7* knockdown inhibited the ability of colony formation in ESCC cells. **(F)** *CDCA7* overexpression promoted the ability of colony formation in ESCC cells. **(G)** *CDCA7* knockdown inhibited the cell cycle of ESCC cells. **(H)** *CDCA7* overexpression promoted the cell cycle of ESCC cells. **(I)** Tumor growth was inhibited significantly in the CDCA7-knockdown group compared with the control group in vivo. Left: tumor tissues in the CDCA7-knockdown group and the control group; right: tumor weight and tumor growth curve. (0.01 < P ≤ 0.05, *; 0.001 < P ≤ 0.01, **; P ≤ 0.001,***).

To verify the biological roles of *CDCA7* in ESCC, we first analyzed the mRNA and protein expression levels in immortal embryonic esophageal epithelium cell lines NE3 and HET-1A and ESCC cell lines including KYSE150, KYSE180, KYSE450, and TE-1 *via* quantitative real-time PCR (q-RTPCR) and Western blot (**Figure S3**). In all these cell lines, we selected KYSE150 and KESE450 as relatively high endogenous *CDCA7* level cell lines for knockdown experiments. KYSE180 was selected as low endogenous *CDCA7* level cell line for overexpression. The efficiency of overexpression and knockdown were verified by Western blot, respectively (**Figures 2A, B**). Then, we detected the changes in cell phenotypes, including proliferation, colony formation, and cell cycle. The results showed that *CDCA7* silencing significantly inhibited the ability of cell proliferation and colony formation in KYSE150 and KESE450 (**Figures 2C, E**), while overexpression of *CDCA7* increased the ability of cell proliferation and colony formation markedly (**Figures 2D, F**). Meanwhile, the results of flow cytometry indicated that *CDCA7* overexpression decreased the proportion of G1-phase cells and increased the proportion of G2 + M-phase cells (**Figure 2H**). On the contrary, *CDCA7* silencing significantly increased the proportion of G1-phase cells and decreased the proportion of G2 + M-phase cells (**Figure 2G**).

To confirm the oncogenic role of *CDCA7 in vivo*, we established a subcutaneous transplantation tumor model in female NU-Foxn1nu nude mice using stable *CDCA7*-knockdown KYSE150 and KYSE150 cells. Four weeks later, tumors were stripped after the mice were sacrificed. The tumor growth rate of the KYSE150 group was significantly faster than that of the *CDCA7*-knockdown group (**Figure 2I**). The results found that the mean tumor volume of the *CDCA7*-knockdown group and the control group was 158.74 ± 24.83 and 488.41 ± 35.84 mm^3^, respectively (*t*-test, *P* < 0.001, **Figure 2I**). The mean tumor weight of the control group and the *CDCA7*-knockdown group was 209.61 ± 108.84 and 54.23 ± 19.39 mg, respectively (*t*-test, *P* < 0.001, **Figure 2I**).

### Cyclins Were Identified as *CDCA7* Targets by ChIP-Sequencing

Immunofluorescence assay was performed in KYSE150, KYSE450, and KYSE180 cells to affirm whether *CDCA7* expresses in the nucleus as CDCA7 was found to be a DNA-binding protein and can serve as a transcription regulator (9, 11, 14). The results showed that CDCA7 was located in both the cytoplasm and the nucleus (**Figure S4**).

**Figure 3:**
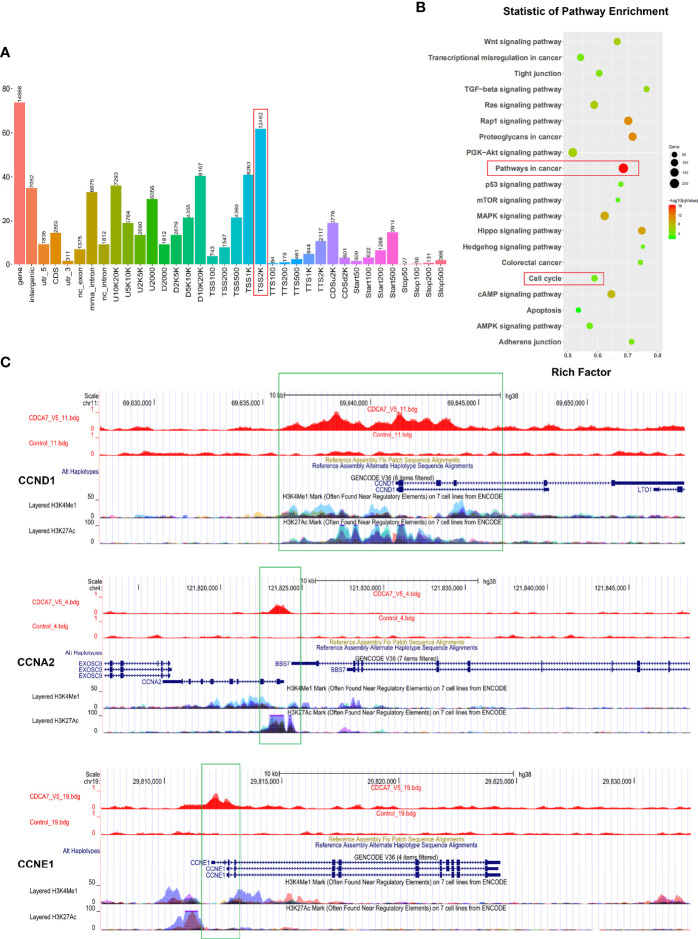
Cyclins may be the downstream genes regulated by CDCA7 as a transcription regulator. **(A)** Most of the binding sites of CDCA7 with DNA located at the position of 2,000 bp before the transcription initiation site of the genes. **(B)** The pathway enrichment analysis of the DNA fragments obtained from ChIP-sequencing. **(C)** CDCA7 binds with *CCND1* at the position of −3,732 to −2,502 bp, binds with *CCNA2* at the position of −74 to 734 bp, and binds with *CCNE1* at the positon of −53 to 925 bp from each transcription start site, respectively.

Since CDCA7 may act as a transcription regulator, chromatin immunoprecipitation sequencing (ChIP-seq) technology was applied to screen a wide range of DNA fragments interacting with CDCA7. Genome-wide mapping of CDCA7-binding profile by ChIP-seq identified 14,930 binding events (*P* < 10^−3^), associated with 11,897 unique genes following a nearest gene annotation. As shown in **Figure 3A**, most (12,462/14,930) of the binding events occur at a distance about 2,000 bp from the transcriptional start site (TSS) of genes, which is generally considered to be the gene promoter region and activation region. These results suggested that CDCA7 may play a role as a transcription factor or transcription regulatory factor. Next, we performed a KEGG pathway enrichment analysis on the 11,897 unique genes which were associated with the DNA fragments obtained from ChIP-seq. The KEGG pathway enrichment analysis showed that target genes were enriched in the pathways including pathways in cancer, cell cycle pathway, PI3K–Akt signaling pathway, MAPK signaling pathway, Ras signaling pathways, and Hippo signaling pathways, which may contribute to ESCC cell proliferation and tumorigenesis (**Figure 3B**). Among the DNA fragments obtained from ChIP-seq, some of them were located in the promoter region of the cell cycle related genes, i.e., 3,000 bp before the transcription initiation site. These genes include *CCND1*, *CCNE1*, *CCNA2*, etcetera (**Figure 3C**). Bdg files, such as the CDCA7_V5.bdg and Control.bdg shown in **Figure 3C**, are the corresponding bedgraph format track files provided by the company, which are convenient to view the position distribution of reads on the genome under different resolution conditions. CDCA7_V5.bdg represents the DNA fragments that can bind to CDCA7 detected by the anti-V5 antibody, and Control.bdg is the DNA fragments of the input group. When we opened the bdg files in the UCSC database and compared CDCA7_V5.bdg with Control.bdg, the location of the peak is the binding site of CDCA7 with the three cyclins. As shown in **Figure 3C**, CDCA7 binds with *CCND1* at the position of −3,732 to −2,502 bp, binds with *CCNA2* at the position of −74 to 734 bp, and binds with *CCNE1* at the positon of −53 to 925 bp from each transcription start site, respectively. At the same time, when the binding of H3K4Me1 and H3K21Ac was displayed on the genome in the UCSC database, we found that apart from *CCNE1*, CDCA7, and H3K4Me1, H3K27Ac shared the same binding position in *CCND1* and *CCNA2* genomes. These findings once again suggested that CDCA7 may play a role as a transcription factor or transcription regulator to regulate the expression of cyclins.

### *CCNA2* May Be the Downstream Target Gene of *CDCA7*

**Figure 4:**
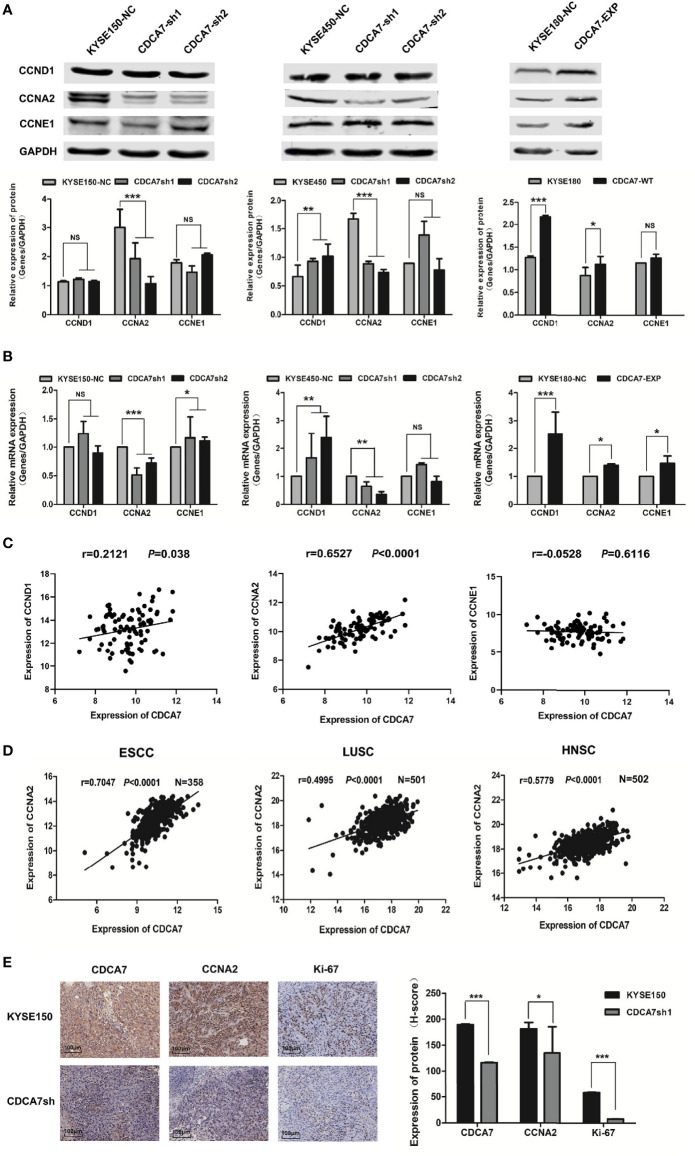
*CCNA2* may be the downstream target gene of CDCA7. **(A)** The protein expression levels of *CCND1*, *CCNA2*, and *CCNE1* in *CDCA7* knockdown cell lines and *CDCA7* overexpression cell lines. **(B)** The mRNA expression levels of *CCND1*, *CCNA2*, and *CCNE1* in *CDCA7* knockdown cell lines and *CDCA7* overexpression cell lines. **(C)** *CDCA7* was positively correlated with *CCND1* expression (*r* = 0.2121, *P* = 0.038) and *CCNA2* expression (*r* = 0.6527, *P* < 0.0001), while it was not correlated with *CCNE1* expression (*r* = −0.0528, *P* = 0.6116). **(D)** The correlation of *CDCA7* and *CCNA2* expression in ESCC, LUSC, and HNSC; correlation coefficient (*r*) and *P*-values were shown in the figures. *P < *0.05 was considered statistically significant. **(E)** IHC assay showed *CDCA7*, *CCNA2*, and *Ki-67* expression in *CDCA7* knockdown xenograft tumor tissue and the control group tissue. Scale bar = 100 μm (P > 0.05, NS; 0.01 < P ≤ 0.05, *; 0.001 < P ≤ 0.01,**; P ≤ 0.001,***).

Since the results of ChIP-seq showed that CDCA7 may act as a transcription regulator to regulate the expression of cyclins, we detected the mRNA and protein levels of the three cyclins in *CDCA7* overexpression and knockdown stable cell lines. We found that the expression level of the *CCNA2* was significantly decreased when *CDCA7* was knocked down and vice versa (**Figures 4A, B**). Meanwhile, we verified the correlation between *CDCA7* and the three cyclins using the mRNA data of 96 ESCC tissues in the TCGA database. The results (**Figure 4C**) showed that there was a weak positive correlation between *CDCA7* and *CCND1* (*r* = 0.2121, *P* = 0.038) and a strong positive correlation between *CDCA7* and *CCNA2* (*r* = 0.6527, *P* < 0.0001), while there was no correlation between *CDCA7* and *CCNE1* (*r* = −0.0528, *P* = 0.6116). Next, we analyzed the mRNA expression data of ESCC (*n* = 358) in GSE53625. Based on the mRNA expression data of ESCC (*n* = 358), *CDCA7* was positively correlated with *CCNA2* (*r* = 0.7047, *P* < 0.0001). Interestingly, when the mRNA expression data of other types of SCC in the TCGA database were analyzed, we found that *CDCA7* was positively correlated with *CCNA2* in LUSC (*n* = 501, *r* = 0.4995, *P* < 0.0001) and HNSC (*n* = 502, *r* = 0.5771, *P* < 0.0001) (**Figure 4D**). Immunohistochemistry was further used to detect the tumor tissue stripped from nude mice with anti-CDCA7, anti-CCNA2, and anti-Ki-67 antibodies. The results showed that the staining intensity of CCNA2 and Ki-67 in the *CDCA7* knockdown group was obviously weaker than that of the KYSE150 group (**Figure 4E**). The *H*-score of CCNA2 and Ki-67 in *CDCA7*-knockdown group (181.344 ± 17.549 and 7.84 ± 0.200) was significantly lower than that in the control group (91.19 ± 9.07 and 58.59 ± 0.626) (*t*-test, *P* < 0.001, **Figure 4E**).

These results revealed that the *CDCA7* probably affected the cell cycle progression, occurrence, and development of cancers through regulating the expression of *CCNA2*.

### *CDCA7* Regulates *CCNA2* Expression Through Binding to the Target Regions of *CCNA2*

**Figure 5:**
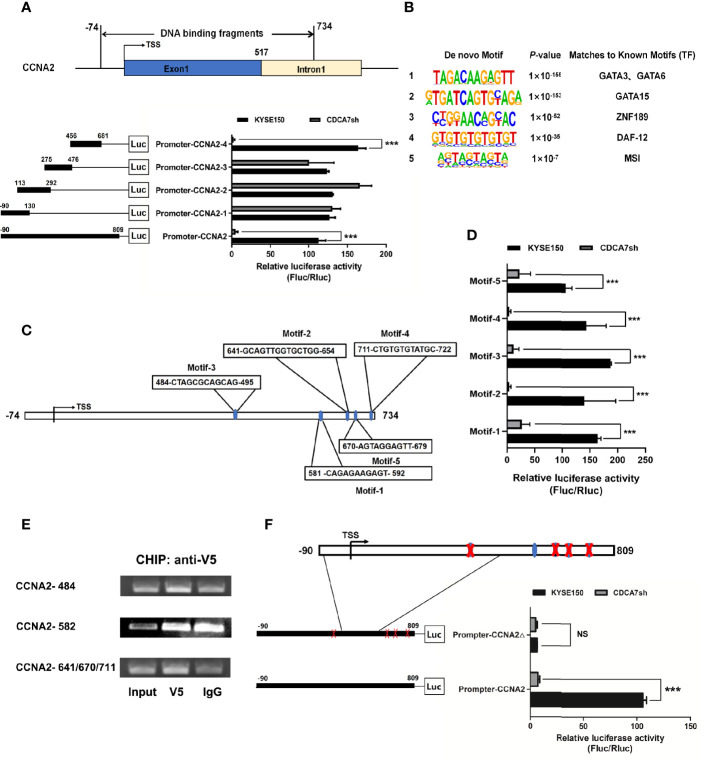
CDCA7 directly binds to *CCNA2* and increases its transcription activity in ESCC cells. **(A)** CDCA7 binds with *CCNA2* from −74 to 734 bp in the ChIP-seq and luciferase reporter assays showed that 456 to 809 bp of *CCNA2* were the core regions regulated by *CDCA7*. **(B)** Five predicted motifs were analyzed from the DNA-binding fragments obtained from the ChIP-seq. **(C)** Five DNA fragments in the 456–809-bp region were highly similar to motif-1, motif-2, motif-3, motif-4, and motif-5. **(D)** Luciferase reporter assays showed that CDCA7 may regulate the transcription of the target genes through the five motifs. **(E)** ChIP-PCR showed that CDCA7 binds to the *CCNA2* at the position of 484–495, 641–654, 670–679, and 711–722 bp in ESCC cells. **(F)** Luciferase reporter assays showed that CDCA7 could not activate the expression of downstream reporter gene when the four binding sites of 484–495, 641–654, 670–679, and 711–722 bp were knocked out. (P > 0.05, NS; P ≤ 0.001,***).

The binding region of CDCA7 on *CCNA2* started from position −74 to 734 bp relative to the TSS, and we constructed the −90 to 809 bp into the pGL3 promoter vector for the dual-luciferase assay subsequently. The reason why we expanded the region of the DNA fragment is the high GC content of the DNA sequence near 734 bp and there is no way to design a suitable pair of PCR primers. To explore the binding domain of CDCA7, the interval from −83 to 809 bp was divided into four segments randomly. The four segments were −90 to 130, 113–292, 275–476, and 456–809 bp. Dual-luciferase assay indicated that the 456–809-bp region of the *CCNA2* was the core element regulated by CDCA7 (**Figure 5A**). To understand the molecular mechanism for the activity of CDCA7 in regulating gene transcription, a *de novo* search for DNA-enriched motifs was performed within the binding fragments and five predicted motifs were obtained. Each of the motifs corresponded to some transcription factor at different degrees (**Figure 5B**). To demonstrate whether CDCA7 acts its role as a transcription factor or a transcription regulator factor by binding with DNA through these motifs, we constructed the sequences of the motifs into pGL3-promoter vectors for dual-luciferase reporting experiments, the results revealed that motif-1 (5′-TAGACAAGAGTT-3′), motif-2 (5′-GTGATCAGTGCAGA-3′), motif-3 (5′-CTGGAACAGCAC-3′), motif-4 (5′-GTGTGTGTGTGT-3′), and motif-5 (5′-AGTAGTAGTA-3′) might be the functional binding sites of CDCA7 with DNA (**Figure 5D**). Next, we compared these motifs with the sequences from −74 to 734 bp and found five DNA fragments highly similar to the five motifs located in the 456–809-bp region (**Figure 5C**). We inferred that the five DNA segments may be the functional binding sites of CDCA7 with *CCNA2*. In order to further research whether CDCA7 directly binds to *CCNA2* through these sites, ChIP-PCR was performed. We found that CDCA7 bound to the DNA fragment from 484 to 495, 641 to 654, 670 to 679, and 711 to 722 bp in the *CCNA2* genomic region (**Figure 5E**). To identify whether CDCA7 regulates *CCNA2* through these binding sites, we constructed a dual-luciferase reporter plasmid with the segments of *CCNA2* from −90 and 809 bp which deleted the 484–495, 641–654, 670–679, and 711–722 bp. The results showed that CDCA7 could not activate the expression of downstream reporter gene when the four binding sites were knocked out (**Figure 5F**). It proved again that CDCA7 may regulate the transcription and expression of *CCNA2* by binding with these four binding sites.

### *CDCA7* Promotes Cell Cycle Through Regulating *CCNA2*

**Figure 6:**
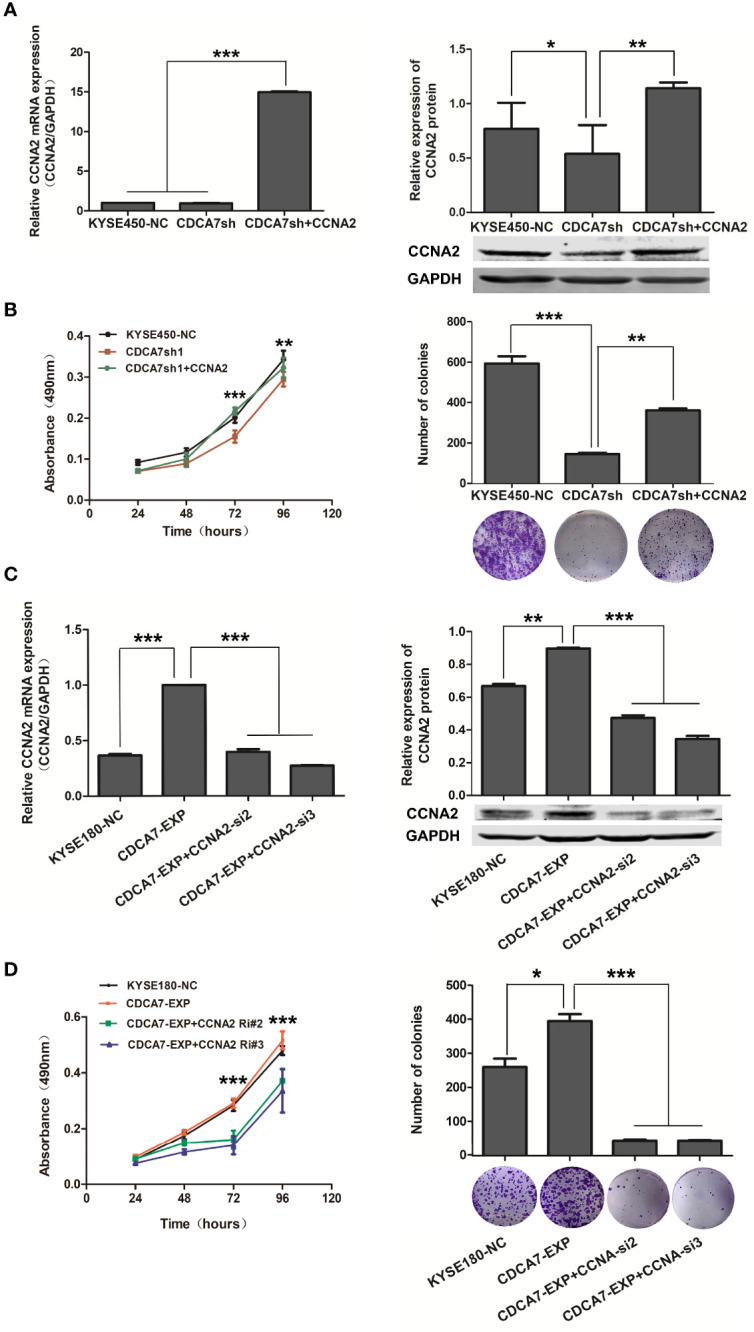
*CDCA7* promotes S-phases *via* transcriptionally regulating *CCNA2* expression. **(A)** *CCNA2* overexpression in KYSE450 cell line with *CDCA7* knockdown; the mRNA and protein expression levels of *CCNA2* and *GAPDH* were detected by qRT-PCR and Western blot. *GAPDH* was used as a loading control. **(B)** *CCNA2* overexpression promoted the proliferation induced by *CDCA7* knockdown. **(C)** *CCNA2* knockdown in KYSE180 cell line with *CDCA7* overexpression; the mRNA and protein expression levels of *CCNA2* and *GAPDH* were detected by Western blot. *GAPDH* was used as a loading control. **(D)** *CCNA2* knockdown inhibited the proliferation induced by *CDCA7* overexpression. (0.01 < P ≤ 0.05, *;0.001 < P ≤ 0.01,**,P ≤ 0.001,***).

To confirm whether *CDCA7* promotes cell cycle through *CCNA2*, we carried out the interference and rescue experiment of *CCNA2*. The results showed that forced overexpression of *CCNA2* in *CDCA7* knockdown ESCC cells (**Figure 6A**) was performed, and a series of phenotype changes had been identified in ESCC cells. The results showed that *CCNA2* overexpression promoted the proliferation and colony formation (**Figure 6B**) induced by *CDCA7* knockdown in KYSE450 cells. Meanwhile, when we silenced its expression in *CDCA7* overexpression ESCC cells (**Figure 6C**), the results of cell phenotype experiments showed that knockdown of *CCNA2* can reduce cell proliferation and colony formation ability (**Figure 6D**) induced by *CDCA7* overexpression in KYSE180 cells. These results suggested that the acceleration effect of *CDCA7* on cell cycle may depend on its transcription regulation of *CCNA2*, and *CCNA2* inhibition may partially reverse the cell proliferation progression induced by *CDCA7* overexpression.

The results indicated that *CDCA7* gene might act as a tumor promoter in ESCC and its copy number amplification or increased expression may accelerate the cell cycle process and promote cell proliferation by binding to the genome of *CCNA2* functional domain and increasing its expression in ESCC. When *CDCA7* is knocked down or decreased, its transcription regulation effect is attenuated, and the cell cycle process and the cell proliferation of ESCC are inhibited as the expression of *CCNA2* is depressed. Furthermore, the mechanism that *CDCA7* acts as an oncogene possibly through regulation of cell proliferation might be applied in various types of SCC.

## Discussion

Previous reports showed that overexpression of *CDCA7* predicts poor prognosis and tumor progression in human breast cancer, lung adenocarcinoma and lymphoma, colorectal cancer, and pancreatic diseases (29–33). In this study, we uncovered the potential prognostic value of *CDCA7*, one of the copy number altered genes, for ESCC patients; revealed the tumor-promoting role of *CDCA7* gene; and explored its possible mechanism in ESCC for the first time. *CDCA7* was highly expressed in not only ESCC but also SCC in transcriptome sequencing data. The Kaplan–Meier survival analysis showed that patients with high expression level of *CDCA7* had poor prognosis. This result reminded us that *CDCA7* may be used as a candidate target to guide the individual diagnosis and a biomarker to establish a technical system for the molecular classification of ESCC.

Further functional studies reveal that CDCA7 may exert its oncogenic roles *via* directly binding to the position of 484–495, 641–654, 670–679, and 711–722 bp from the transcription start site of *CCNA2*. The data of TCGA and GSE53625 further confirmed the positive correlation between *CDCA7* and *CCNA2* in ESCC, indicating that the high expression level of *CDCA7* may be an important driving event in the occurrence and development in ESCC.

*CCNA2*, which is one of the two A-type cyclins and ubiquitously expressed in cultured cells, has been reported to be upregulated in a variety of cancers (34–37). CCNA2 is considered to be the critical S-phase cyclin in mammalian cells (18, 38). *CCNA2* is expressed at the beginning of the S-phase (16, 39) and existed in both the S- and G2-phases. Once synthesized, it binds with its catalytic partners, the cyclin-dependent kinases (CDK) CDK2 and CDK1, and activates its catalytic activity. The CDK2/CCNA2 complex promotes DNA replication through localizing to replication foci in the nucleus (17, 40). The complexes phosphorylate the proteins which play important roles in DNA synthesis and thus drive the S-phase progression (16, 18, 19, 41–43). In addition, a second function of CCNA2 is involved in the entry of cells into mitosis since it also is expressed at the G2-phase (44). The accumulation of CCNA2 is rate-limiting for S-phase entry, so overexpression of *CCNA2* can induce cultured cell early entry into the S-phase under normal circumstances (45, 46). Indeed, inhibition of CCNA2 function by p21Cip1 during the G2-phase or injection of anti-CCNA2 antibodies into cultured fibroblasts both can block the process of cells into mitosis (41, 47).

It is known to all that disorder of the cell cycle process is one of the causes of many cancers (48–52). Cancer cells lose many of the inhibitory controls in the cell cycle because of the inactivation or mutation of suppressor genes and overexpression or amplification of oncogenes (53). The aberrant transcription of upregulation of cyclins and CDKs can result in uncontrolled cell cycle progression and mitosis. Our study showed that *CCNA2* was a direct downstream target gene of CDCA7, and its expression may be activated by CDCA7 on both the transcription level and the protein level. Therefore, the copy number amplification or increase of *CDCA7* may lead to a high level expression of *CCNA2* to accelerate the cell cycle process. This may be a mechanism and indicate the important role of *CDCA7* in ESCC. Therefore, we speculated that patients with high expression of *CDCA7* could be treated with cell cycle-specific agents (CCSA) since the expression of *CCNA2* and the number of cells in the proliferative phase are correspondingly increased. This study provides a theoretical and experimental foundation for the research and development of drug targets for clinical treatment of ESCC in China.

In summary, our study shows that *CDCA7*, a copy number amplification gene in ESCC, may act as a tumor promoter *via* regulating *CCNA2* directly and accelerate the cell cycle process of ESCC cells. The copy number amplification may lead to tumorigenesis and progression of ESCC. Our findings provide a new insight into the molecular mechanisms involved in ESCC development. However, there are still some deficiencies in our research process. Whether the high expression level of *CDCA7* is more sensitive to CCSA as we expected needs further experimental verification. Meanwhile, further in-depth research is needed to clarify the mechanism of ECSS carcinogenesis, to develop the prognostic method, and to identify feasible therapeutic targets which could be used to overcome the disease.

## Data Availability Statement

Publicly available datasets were analyzed in this study. These data can be found here: https://www.ncbi.nlm.nih.gov/geo/query/acc.cgi?acc=GSE53625, https://xenabrowser.net/, and https://www.cbioportal.org/.

## Ethics Statement

The studies involving human participants were reviewed and approved by the Institutional Reviewing Board (IRB) and the Research Committee of Shanxi Medical University. The patients/participants provided their written informed consent to participate in this study. The animal study was reviewed and approved by Institutional Reviewing Board (IRB) and the Laboratory Animal Welfare Committee of Shanxi Medical University.

## Author Contributions

YC and XC designed the study. HL, YJW, SJW, FW, CC, EX, SQW, DG, FC, YB, and YM acquired the data. HL, YJW, SJW, and HC analyzed the data. HL prepared the manuscript. PK, YQW, and LZ edited the manuscript. YC reviewed the manuscript. All authors contributed to the article and approved the submitted version.

## Funding

The work was supported by funds from the National Natural Science Foundation of China (81602175, 81773150, 81972613), the Fund for Shanxi “1331 Project” and “1331 Project” Key Subjects Construction, the Fund for “Sanjin Scholars,” the Foundation for Youths of Shanxi Province (201901D211349, 201901D211345), and the Natural Science Foundation of Shanxi Province (201801D121306).

## Conflict of Interest

The authors declare that the research was conducted in the absence of any commercial or financial relationships that could be construed as a potential conflict of interest.

## Publisher’s Note

All claims expressed in this article are solely those of the authors and do not necessarily represent those of their affiliated organizations, or those of the publisher, the editors and the reviewers. Any product that may be evaluated in this article, or claim that may be made by its manufacturer, is not guaranteed or endorsed by the publisher.
