# Supplementary material for: CDCA7 Facilitates Tumor Progression by Directly Regulating CCNA2 Expression in Esophageal Squamous Cell Carcinoma
Source: Front Oncol. 2021 Oct 19;11:734655. doi: 10.3389/fonc.2021.734655 (PMC8561731; doi:10.3389/fonc.2021.734655)
Supplement: Supplementary file 1 [file DataSheet_1.docx]

**Table S1. The primers used for ChIP-PCR**

|  | Forward | Reverse |
| --- | --- | --- |
| CCNA2-484 | AGCAGTGATGTTGGGCAACTCTG | CATCCCTTTACCCGTCTCGTCTT |
| CCNA2-582 | GCAGGGTTTGGCATTGGCTTAG | CAAACCACACACCAGCACCAAC |
| CCNA2-641/670/711 | AAGACGAGACGGGTAAAGGGATG | GGCGGGGTCCTGATGCTAATATG |

**Table S2. The primers used for Real time-PCR**

|  | Forward | Reverse |
| --- | --- | --- |
| *CDCA7* | CTTGTCATCAATGCCGTCAG | CAGTTGCAGATTCCTCGACA |
| *CCND1* | CCCGCACGATTTCATTGAAC | AGGGCGGATTGGAAATGAAC |
| *CCNA2* | CAGTGAGTGTTAATGAAGTACCAG | CCTATCAATGTAGTTCACAGCC |
| *CCNE1* | CGGTATATGGCGACACAAGA | AGGGGACTTAAACGCCACTT |
| *GAPDH* | GAAGGTGAAGGTCGGAGTC | GAAGATGGTGATGGGATTTC |

**Table S3. The primers used for construction of dual-Luciferase reporter plasmids**

|  | Forward | Reverse |
| --- | --- | --- |
| DNA fregement:  -90-809 | CGGGGTACCCCCCTGCTCAGTTTCCTTTG | GGAAGATCTGGAGCTGAG  CGAAGACTACAC |
| DNA fregement:  -90-130 | CGGGGTACCCCCCTGCTCAGTTTCCTTTG | GGAAGATCTGAGGAGGTTGCGAAAGGC |
| DNA fregement:  113-292 | CGGGGTACCGCCTTTCGCAACCTCCTC | GGAAGATCTGAGTGGACGGCGGGATC |
| DNA fregement:  275-476 | CGGGGTACCGATCCCGCCGTCCACTC | GGAAGATCTGGTTCCCGGACTTCAGTACC |
| DNA fregement:  456-681 | CGGGGTACCGGTACTGAAGTCCGGGAACC | GGAAGATCTGAACTCCTACTGTGGCAAACC |

**Figure S1.**

**
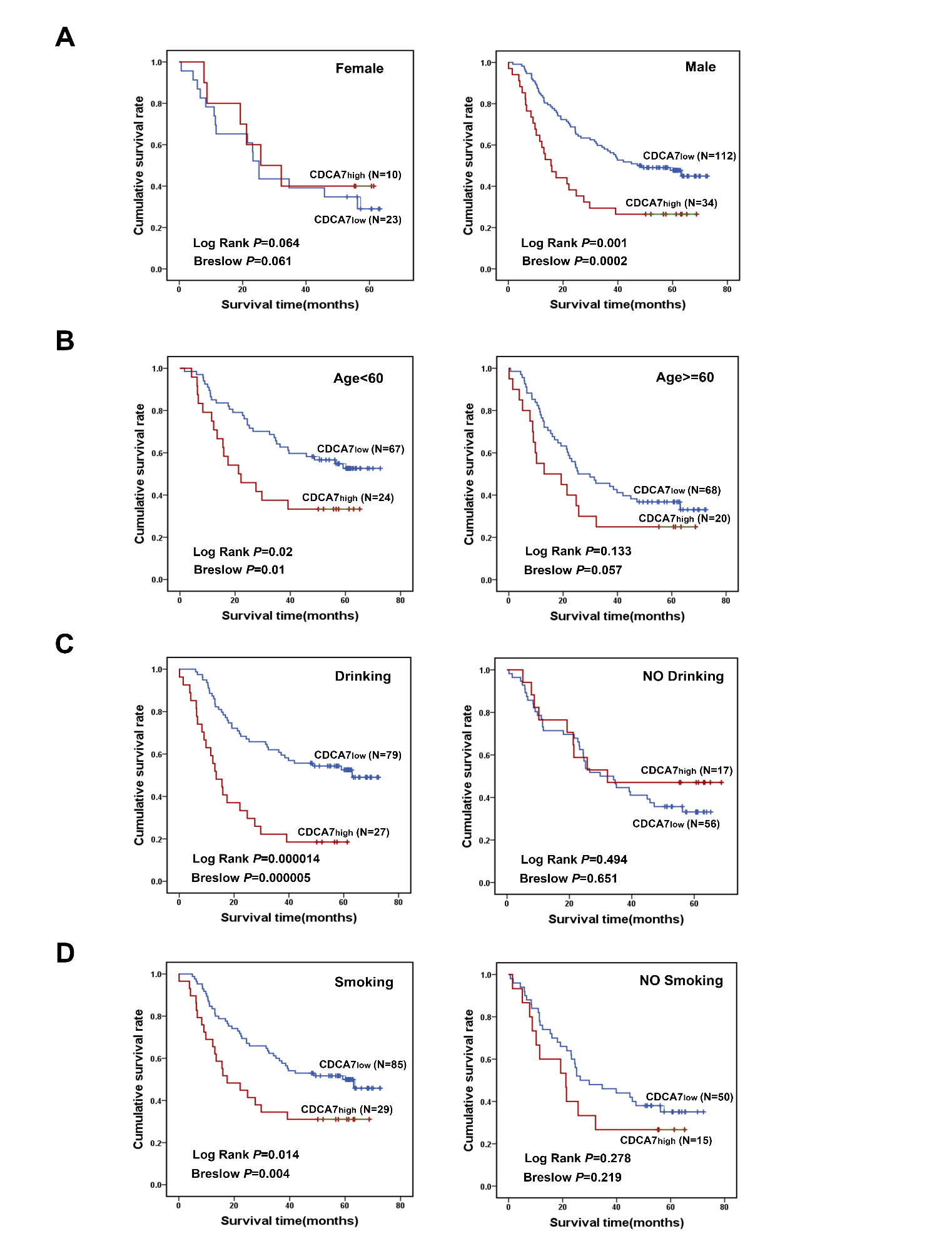
**

**Figure S1. Kaplan-Meier survival plot showed the clinical value of *CDCA7* expression in ESCC patients with different gender, age, different smoking status and drinking status. (A)** Kaplan-Meier survival plot showed the cumulative survival rate of ESCC patients with different *CDCA7* expression in male and female groups. **(B)** Kaplan-Meier survival plot showed the cumulative survival rate of ESCC patients with different *CDCA7* expression in Age<60 and Age≥60 groups. **(C)** Kaplan-Meier survival plot showed the cumulative survival rate of ESCC patients with different *CDCA7* expression in Drinking and NO Drinking groups. **(D)** Kaplan-Meier survival plot showed the cumulative survival rate of ESCC patients with different *CDCA7* expression in Smoking and NO Smoking groups. Log rank test and Breslow test were used to analyze the survival data of ESCC patients with different gender, age, different smoking status, drinking status. And P values were showed in the figures. *P* < 0.05 was considered statistically significant.

**Figure S2.**

**
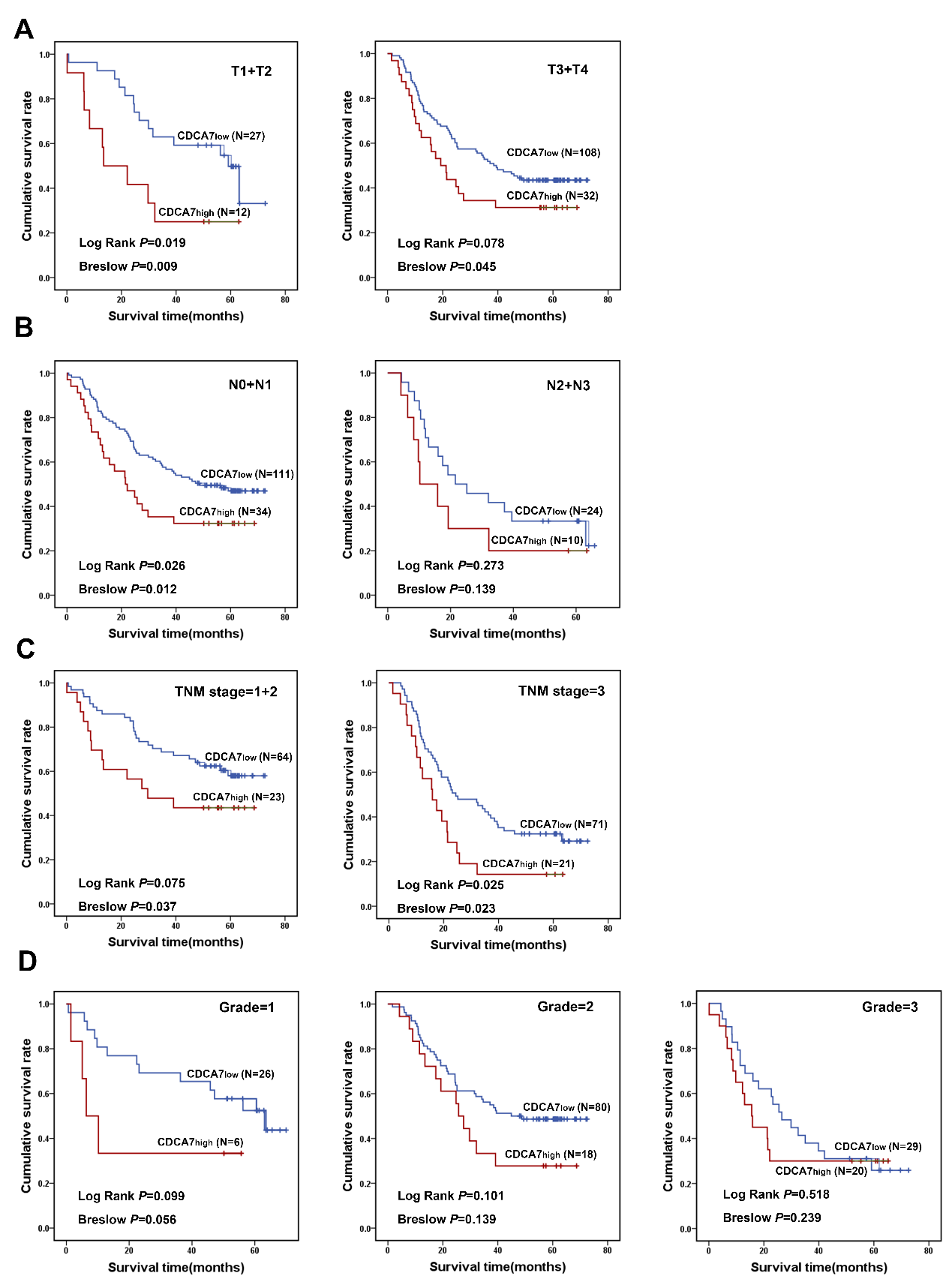
**

**Figure S2. Kaplan-Meier survival plot showed the clinical value of *CDCA7* expression in ESCC patients with different T stage, N stage, TNM stage and Grade. (A)** Kaplan-Meier survival plot showed the cumulative survival rate of ESCC patients with different *CDCA7* expression in T1+2 and T3+4 groups. **(B)** Kaplan-Meier survival plot showed the cumulative survival rate of ESCC patients with different *CDCA7* expression in N(0-1) and N(2-3) groups.. **(C)** Kaplan-Meier survival plot showed the cumulative survival rate of ESCC patients with different *CDCA7* expression in TNM I+II and TNM III groups. **(D)** Kaplan-Meier survival plot showed the cumulative survival rate of ESCC patients with different *CDCA7* expression in Grade1, Grade2 and Grade3 groups. Log rank test and Breslow test were used to analyze the survival data of ESCC patients with different gender, age, different smoking status, drinking status. And P values were showed in the figures. *P* < 0.05 was considered statistically significant.

**Figure S3.**

**
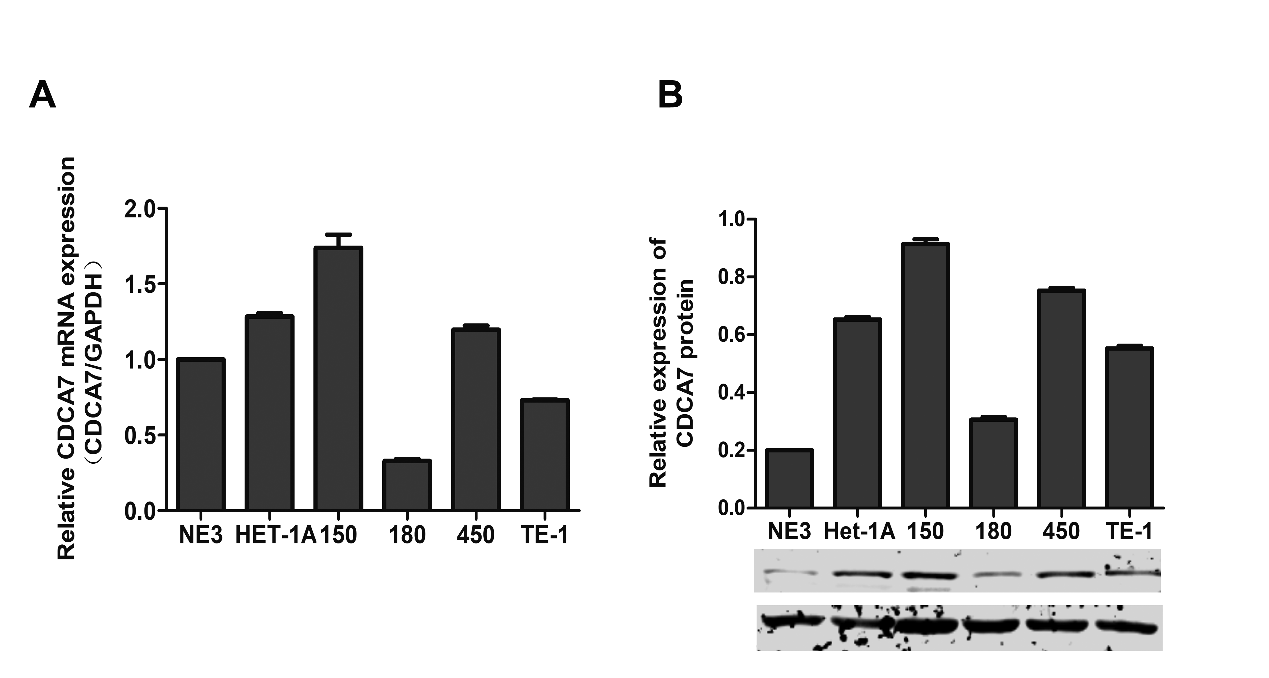
**

**Figure S3. *CDCA7* expression levels in different ESCC cell line. (A)** *CDCA7* mRNA expression levels. **(B)** CDCA7 protein expression levels.

**Figure S4.**

**
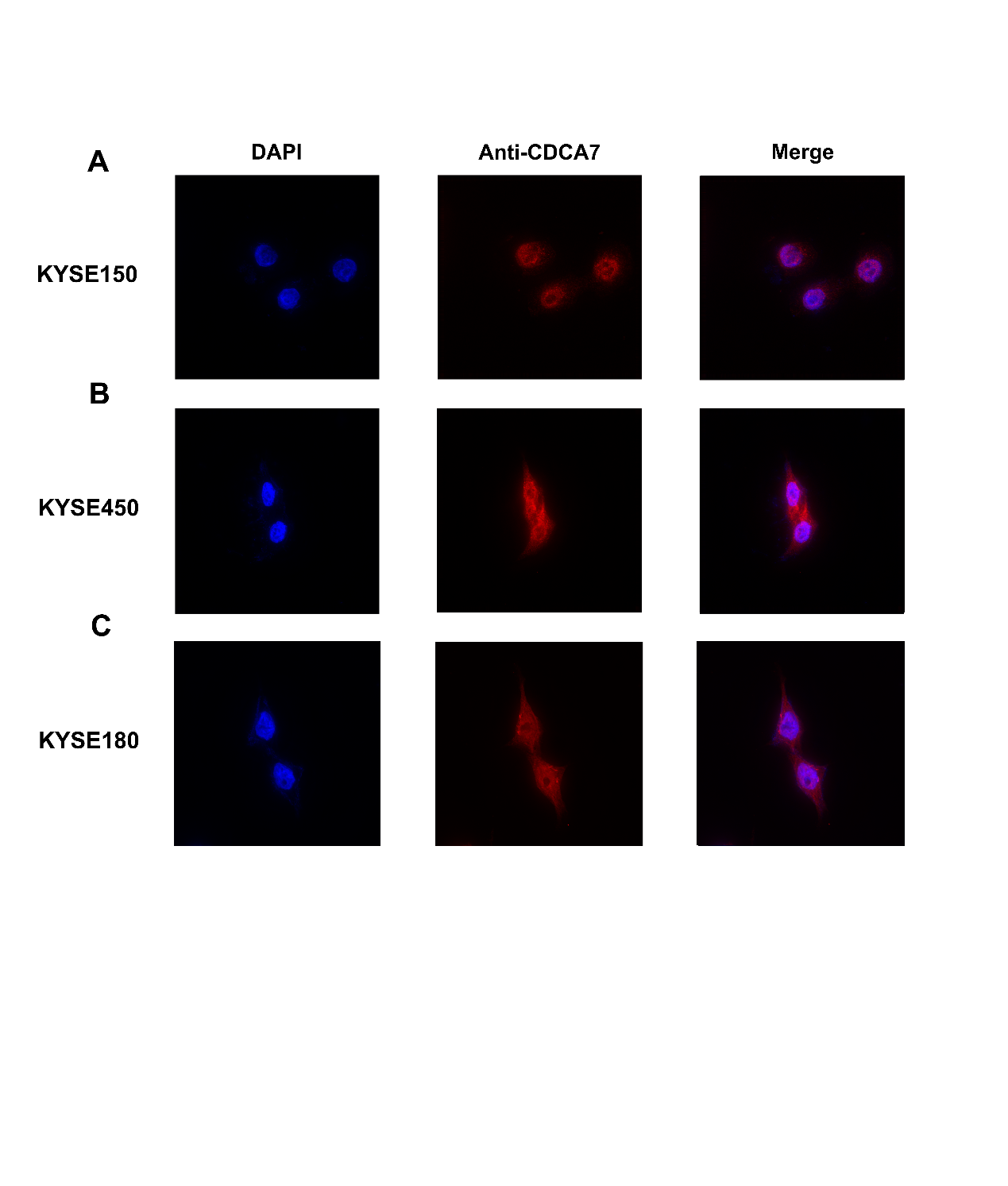
**

**Figure S4. Immunofluorescence images of ESCC cells with V5-tagged *CDCA*7**

**Overexpression. (A)** The immunofluorescence images of ESCC cells KYSE150. **(B)** The immunofluorescence images of ESCC cells KYSE450. **(C)** The immunofluorescence images of ESCC cells KYSE180.
